# Cross‐Taxa Analysis of Long‐Term Data Reveals a Positive Biodiversity‐Stability Relationship With Taxon‐Specific Mechanistic Underpinning

**DOI:** 10.1111/ele.70003

**Published:** 2025-04-03

**Authors:** Arthur V. Rodrigues, Tuuli Rissanen, Mirkka M. Jones, Ida‐Maria Huikkonen, Otso Huitu, Erkki Korpimäki, Mikko Kuussaari, Aleksi Lehikoinen, Andreas Lindén, Hannu Pietiäinen, Juha Pöyry, Pasi Sihvonen, Anna Suuronen, Kristiina Vuorio, Marjo Saastamoinen, Jarno Vanhatalo, Anna‐Liisa Laine

**Affiliations:** ^1^ Research Centre for Ecological Change, Organismal and Evolutionary Biology Research Programme, Faculty of Biological and Environmental Sciences University of Helsinki Helsinki Finland; ^2^ Helsinki Institute of Life Science, University of Helsinki Helsinki Finland; ^3^ Aalto University Espoo Finland; ^4^ Finnish Environment Institute (SYKE) Helsinki Finland; ^5^ Natural Resources Institute Finland (Luke) Helsinki Finland; ^6^ Department of Biology, Section of Ecology University of Turku Turku Finland; ^7^ Finnish Museum of Natural History University of Helsinki Helsinki Finland; ^8^ University of Helsinki Helsinki Finland; ^9^ Department of Mathematics and Statistics, Faculty of Science University of Helsinki Helsinki Finland

**Keywords:** abundance data, asynchrony, diversity–stability relationship, functional traits, long‐term monitoring, species richness, stability

## Abstract

Anthropogenic environmental change is altering biodiversity at unprecedented rates, threatening the stability of ecosystem services on which humans depend. However, most of what is known about biodiversity–stability relationships comes from experimental studies making extrapolation to real ecosystems difficult. Here, we ask whether the shape and underlying mechanisms of the biodiversity–stability relationship vary among taxa in real‐world communities. Our study harnesses the power of six terrestrial and aquatic long‐term monitoring datasets, encompassing entire assemblages at hundreds of georeferenced sites providing 20 years long community measurements, covering a 1200 km latitudinal gradient across Finland. In general, we detect a positive relationship between species richness and stability. Structural equation modelling reveals that this relationship is modified by functional trait community composition, with specific mechanisms varying among the taxa. Our study is among the first to highlight the importance of functional traits in elucidating both general and taxon‐specific impacts of biodiversity on community stability.

## Graphical abstract

We investigate the relationship between biodiversity and stability in real‐world communities, examining if this relationship and its underlying mechanisms differ among various taxa. We utilise six long‐term monitoring datasets from both terrestrial and aquatic environments across a 1200 km latitudinal gradient in Finland, providing two decades of community measurements. We find a positive correlation between species richness and stability, which is influenced by the composition of the functional trait community. However, we note that the specific mechanisms of this relationship vary among taxa. We emphasise the importance of functional traits in understanding the impacts of biodiversity on community stability.

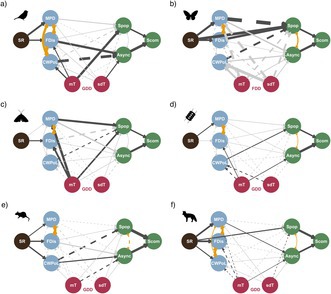

## 1 Introduction

Biodiversity has long been suggested to promote temporal stability of communities (Lehman and Tilman 2000; Thibaut and Connolly 2013) and such a relationship has been demonstrated in a number of theoretical and experimental studies (Cardinale et al. 2012; Hector et al. 1999; Tilman, Reich, and Knops 2006). With multiple anthropogenic pressures on biodiversity, it is increasingly important to understand the relationship between different dimensions of diversity and community stability in natural communities that vary in diversity and are subject to real‐world regimes of environmental disturbance (Donohue et al. 2016). Community stability – the ratio between temporal mean and standard deviation of an aggregate community‐level metric such as abundance – can be decomposed into two interacting components, population stability and asynchrony (Thibaut and Connolly 2013; Zhao et al. 2022), in which both components can promote ecosystem stability (Lehman and Tilman 2000; Gross et al. 2014). Population stability is the average constancy of species' population sizes weighted by their relative abundances in the community, while asynchrony is the lack of synchrony in population fluctuations within a community over time. The mechanisms through which diversity promotes population stability and asynchrony include intraspecific density dependence and interspecific interactions, such as predation and competition (Loreau and de Mazancourt 2013), and variability in species' responses to environmental conditions, which are expected to increase in diverse communities due to niche differentiation or portfolio effects in which species richness indirectly promotes community stability via asynchrony (Tredennick et al. 2017; de Bello et al. 2021).

Theoretical and empirical evidence have suggested that functional traits of organisms in local communities mediate the biodiversity–ecosystem function relationships (Craven et al. 2018; de Bello et al. 2021; Li et al. 2021; Grace, Loreau, and Schmid 2022). There are at least three ways by which functional traits can stabilise communities: by increasing functional diversity, by communities being dominated by stable and often conservative species and by insurance effect, a long‐term change in the species composition due to replacement of species with different response to perturbation but with similar effect on ecosystem functioning (de Bello et al. 2021). Communities with higher functional diversity tend to be more stable because species with different functional traits would respond differently to environmental variability, consequently increasing asynchrony and community stability (de Bello et al. 2021). Since this increase in asynchrony might be driven by multi‐trait responses, phylogenetic diversity can provide information on unmeasured trait variation (Tucker et al. 2018). Of particular interest are traits associated with the pace‐of‐life syndrome (POLS) in which species are positioned along a fast‐slow continuum with slow‐paced species being characterised by long life span at the cost of slow growth and low reproductive rate, whereas fast‐paced species present the opposite trait values (Ricklefs and Wikelski 2002). Therefore, communities dominated by slow‐paced species might show increased population stability because they are more resistant to environmental fluctuations (de Bello et al. 2021). Conversely, communities dominated by fast‐paced species can also result in more stable population, for example when a fast response to environmental perturbation also provides a fast recovery (insurance effect).

Much of the empirical support for a positive relationship between species diversity and community stability comes from biodiversity experiments that manipulate plant species number (Tilman, Reich, and Knops 2006; Craven et al. 2018; Zhao et al. 2022), while studies using observational data and encompassing numerous taxonomic groups are relatively rare (but see Jarzyna et al. 2022, Wisnoski et al. 2023). Moreover, current literature mainly focuses on exploring the effect of taxonomic diversity on stability (Liang et al. 2022, Wisnoski et al. 2023), while studies incorporating other facets of diversity (e.g., functional diversity) tend to focus on a single species group (Craven et al. 2018; Li et al. 2021). Thus, while the importance of functional traits on community assembly and functioning is increasingly recognised (Gross et al. 2017), it remains unknown how ubiquitous the biodiversity–stability relationship is across variable taxa in natural communities and how functional trait diversity contributes to this relationship in the wild (Qiao et al. 2023). To address these knowledge gaps, we harness high‐resolution temporal data for over 900 species representing six taxonomic groups in boreal and subarctic ecosystems in Finland across 20‐year time‐periods. We apply a structural equation modelling framework to quantify (a) the effects of species richness and abiotic environmental drivers on functional diversity and composition (i.e., functional diversity, phylogenetic diversity and community‐weighted mean of pace of life) and (b) the effects of functional diversity and composition, together with environmental drivers, on community stability via impacts on population stability and species asynchrony.

Overall, we predict a positive relationship between species richness and community stability that will be mediated by functional trait responses to environmental fluctuation (de Bello et al. 2021). We expect that communities with higher species richness have higher functional diversity, which in turn results in more stable populations due to lower intraspecific competition and/or increased population asynchrony (Loreau and de Mazancourt 2008, 2013). We also expect that the mechanisms driving diversity–stability relationship may differ among taxa because each taxon occupies a different range of pace‐of‐life‐continuum (i.e., butterfly communities can be overall faster compared to large mammals, for example). Our work highlights how systematically collected long‐term monitoring data are crucial in elucidating both general impacts of biodiversity on community stability across taxa and in generating a more mechanistic understanding of taxon‐specific relationships.

## 2 Materials and Methods

To understand the diversity–stability relationships across taxa, we analyse long‐term, systematically collected, data on bird, butterfly, moth, phytoplankton, small mammal and large mammal communities. First, we explore the bivariate relationships between diversity and stability variables using Spearman correlation. We then fit structural equation models to test whether the pathways through which species richness and stability are associated vary among taxa. In our models, we include species richness, functional dispersion, mean phylogenetic distance and community pace‐of‐life to consider different dimensions of diversity as well as summer and winter conditions to account for the abiotic environment. Finally, to enhance the quantitative explanations of our results, we calculate indirect effects of the variables on community stability.

### 2.1 Study Area and Species Data

**FIGURE 1:**
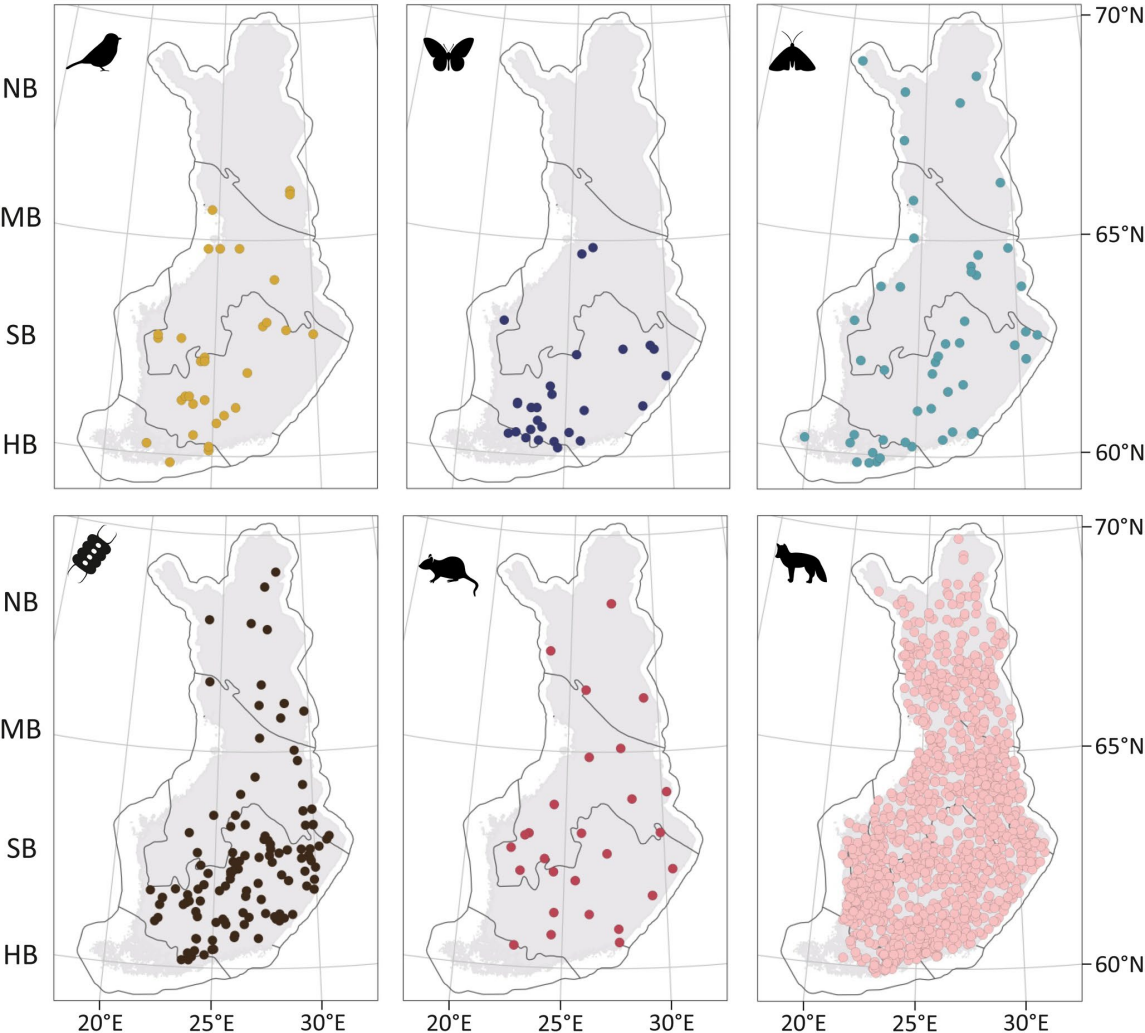
Study sites for each investigated taxon, namely (from left to right): Birds, butterflies, moths, phytoplankton, small mammals, and large mammals. Each dot in small mammals panel represents two sampling sites which are located very close to each other, but in different habitats. The bioclimatic zones are shown in the background and marked with acronyms on the left. HB = hemiboreal, SB = south boreal, MB = mid boreal, NB = north boreal.

Our study area covers a ~1200 km latitudinal gradient (ca 60°–70°N) and four bioclimatic zones across Finland ranging from hemiboreal southern coast to north boreal forests and even subarctic treeless tundra (Figure 1). The polar front and North Atlantic current strongly affect climate in Finland, driving temperature and precipitation patterns and their seasonality (Tikkanen 2005). Along the latitudinal gradient, mean annual temperature varies from ca 7°C in the southwest to −2°C in the north (period 1991–2020, Jokinen et al. 2021).

Across Finland, several taxa have been systematically monitored for national assessment of species' spatiotemporal population trends (Roslin and Laine 2022) following the same protocols as similar monitoring schemes that are carried out across Europe (see e.g., Fox et al. 2013 for moths in United Kingdom and Sevilleja et al. 2019 for Europe‐wide butterfly monitoring). Species' monitoring schemes were established in the 1960–1990s and are replicated annually in a subset of the sites. We used species abundances, reported as the number of individuals (biomass for phytoplankton) to calculate community metrics. The abundances were standardised using trapping effort (e.g., transect length or trapping period, sample volume for phytoplankton), and summed for each year per site. To calculate the functional and phylogenetic diversity metrics we collated species phylogenies and trait data on growth, survival, reproduction, dispersal and resource usage for all taxa by choosing 4–5 traits that represent analogous functions among the different taxa (Table S1). Short descriptions of each dataset are presented below, and descriptive statistics are found in Table 1.

**TABLE 1:** Descriptive statistics of time series data used in the study across different taxonomic groups. Study years represent the full monitoring period within which the investigated sites had data. Sampling frequency reports the minimum, median and maximum frequency of sampling across sites within each taxon. If a site was sampled every year during the 20‐year period, its sampling frequency is 1.

Taxon	Number of sites	Number of species	Study years	Sampling frequency (Min./Median/Max.)
Birds	40	159	1981–2019	0.3/1.0/1.0
Butterflies	27	82	1999–2019	0.35/0.85/1.0
Moths	52	480	1993–2021	0.3/0.9/1.0
Phytoplankton	119	163	1977–1997	0.25/0.4/1.0
Small mammals	50	20	1979–2018	0.65/0.95/1.0
Large mammals	1114	20	1989–2020	0.25/0.75/1.0

### 2.2 Bird Data

Bird observation data are curated by the Finnish Museum of Natural History and have been collected yearly since the 1970s using line transect censuses based on a one‐visit census typically in June following the breeding phenology of birds (Lehikoinen 2013). Hence, monitoring is carried out earlier in southern than in northern Finland. Birds are counted by an observer walking 3–6 km long transects between 3:00 and 9:00 a.m., when the singing activity of birds is highest. The line transect from which birds are observed is 50 m wide, but observations are recorded also from as far as the birds can be detected. Trait data for migratory status, generation length, body mass, feeding and maximum brood were compiled from different sources including bird handbooks and atlases (see full list of refences in Table S2). One hundred phylogenies were randomly selected from a global phylogeny including almost 10,000 extant bird species (Jetz et al. 2012) using R package rtrees (version 1.0.2, Li 2023).

### 2.3 Butterfly Data

We combined two similar butterfly monitoring datasets: a network of volunteer‐based transects established in 1999 by the Finnish Environment Institute (Heliölä, Huikkonen, and Kuussaari 2022) and a survey of standardised transects which ran from 2001 to 2014 (Kuussaari et al. 2007). The volunteers (with professional experience in species identification) walk the transects, length of 1.5–3 km and constant location, annually at least seven times between May and late August. Within transects, the individuals of each species are recorded from a 5 × 5 × 5 m^3^ cube ahead of the observer as they walk the transect (Pollard and Yates 1993; Heliölä, Huikkonen, and Kuussaari 2022). The standardised transects were 1 km long and surveyed by researchers seven times per summer. Trait data for butterflies were derived from a European and Maghreb butterfly trait database (Middleton‐Welling et al. 2020). We used wingspan, overwintering stage (egg, larvae, pupa and adult), voltinism (univoltine, bivoltine and multivoltine) and host plant usage (monophagous, oligophagous and polyphagous). A phylogenetic tree for butterflies was extracted from a complete time‐calibrated multi‐gene consensus phylogeny of 496 extant European butterfly species (Wiemers et al. 2020).

### 2.4 Moth Data

Moth data (including only macromoths) have been collected since 1993 under the National Moth Monitoring scheme, coordinated by the Finnish Environment Institute (Leinonen et al. 2016). Sampling of nocturnal moths is carried out every night from early April to late October in southern Finland and from early May to early October in northern Finland using light traps. The traps are kept in the same location from year to year; however, not all traps are sampled every year. Volunteer lepidopterists with professional‐level experience in species identification empty the traps weekly and identify occurring species, and monitoring coordinators perform data cross‐checking (Pöyry et al. 2011). Trait data for moths were compiled from Finnish Lepidopterology handbooks by researchers at the Finnish Environment Institute and the Finnish Museum of Natural History (Yazdanian et al. 2023). We use the same four traits for moths as for butterflies. A moth phylogeny was derived from a phylogenetic hypothesis created by Pöyry et al. (2017) and updated in Hällfors et al. (2021), which includes over 800 moth species.

### 2.5 Freshwater Phytoplankton Data

Freshwater phytoplankton data were acquired from the Finnish national phytoplankton monitoring database, which is maintained by the Finnish Environment Institute and comprises observations since 1977. Phytoplankton communities are annually surveyed from lake surface water samples taken between July and August (Syke 2024). Phytoplankton abundances are reported using standardised biovolume (μg/L). To match community data with available trait and phylogeny information in Weigel et al. (2022), we used the same 165 species as in their original paper. To characterise the ecological trait structure of these communities, we used cell volume, chain formation, nitrogen fixation and motility.

### 2.6 Small Mammal Data

Small mammal species (i.e., small rodents and shrews) have been inventoried biannually since the 1960s by the Natural Resources Institute Finland (Korpela et al. 2013). Trapping is conducted both in spring (mid‐April to mid‐June) and autumn (mid‐August to mid‐October). The inventory locations are constant, but in some cases the individual trapping sites have changed over time due to land use changes. However, the new trapping sites are selected to be as similar and as close as possible to the earlier sites. Trait data for small mammals were derived from a global coalesced mammal database of intrinsic and extrinsic traits (COMBINE, Soria et al. 2021). Here we used body mass, generation length, litter size, dietary breadth and dispersal distance. From the phylogenetic atlas of mammal macroecology, we randomly selected 100trees (PHYLACINE v1.2, Faurby et al. 2018).

### 2.7 Large Mammal Data

Mammal snow track counts have been monitored systematically since 1989 by the Natural Resources Institute Finland (Lindén et al. 1996). Data are collected using a network of transects shaped as equilateral triangles, with a total length of ca 12 km (each side of the triangle is 4 km). The transects have fixed locations and about half of the sites are visited annually by volunteer experts. All snow tracks of mammal species crossing the transect are recorded, and monitoring is usually carried out between mid‐January and mid‐March (Helle, Ikonen, and Kantola 2016). Trait and phylogenetic information for large mammals were compiled similarly as for small mammals and we also used the same five traits as reported above.

### 2.8 Environmental Variables

Environmental data were obtained from the Finnish Meteorological Institute (FMI 2023). Acquired temperature data are based on spatial interpolation of weather station data at 10‐km resolution (Aalto, Pirinen, and Jylhä 2016). We used daily mean temperatures (period 1961–2022) to calculate growing degree days (GDD) and freezing degree days (FDD) for each study year present in the community data. GDD summarises daily mean temperatures above 5°C and FDD is a sum of daily mean temperatures below 0°C, respectively. We use GDD and FDD to describe summer and winter conditions as they include more information on temperature variation and extremes than annual average temperature. Seasonal variation can also be more strongly linked with the taxonomic and functional structure of the communities (Marjakangas et al. 2022). Furthermore, the variables used capture both environmental and spatial variation across the study sites due to their strong association with latitudinal gradient.

### 2.9 Site Selection

The monitoring data cover a long period of sampling (> 20 years), but sites were not sampled every year during the monitoring period. To guarantee reliability of stability and asynchrony metrics at selected sites, we: (i) identified the fullest sampling period within a 20‐year time frame using a moving window approach; (ii) removed sites with < 5 years sampled; (iii) removed sites with < 15 years of difference between the first and the last sampling event. Hence, the selected time series for each site varied from 15 to 20 years in length and could represent any 20‐year period within the monitoring time series (see Table 1 for the taxon‐specific sampling frequencies).

### 2.10 Statistical Analyses

**FIGURE 2:**
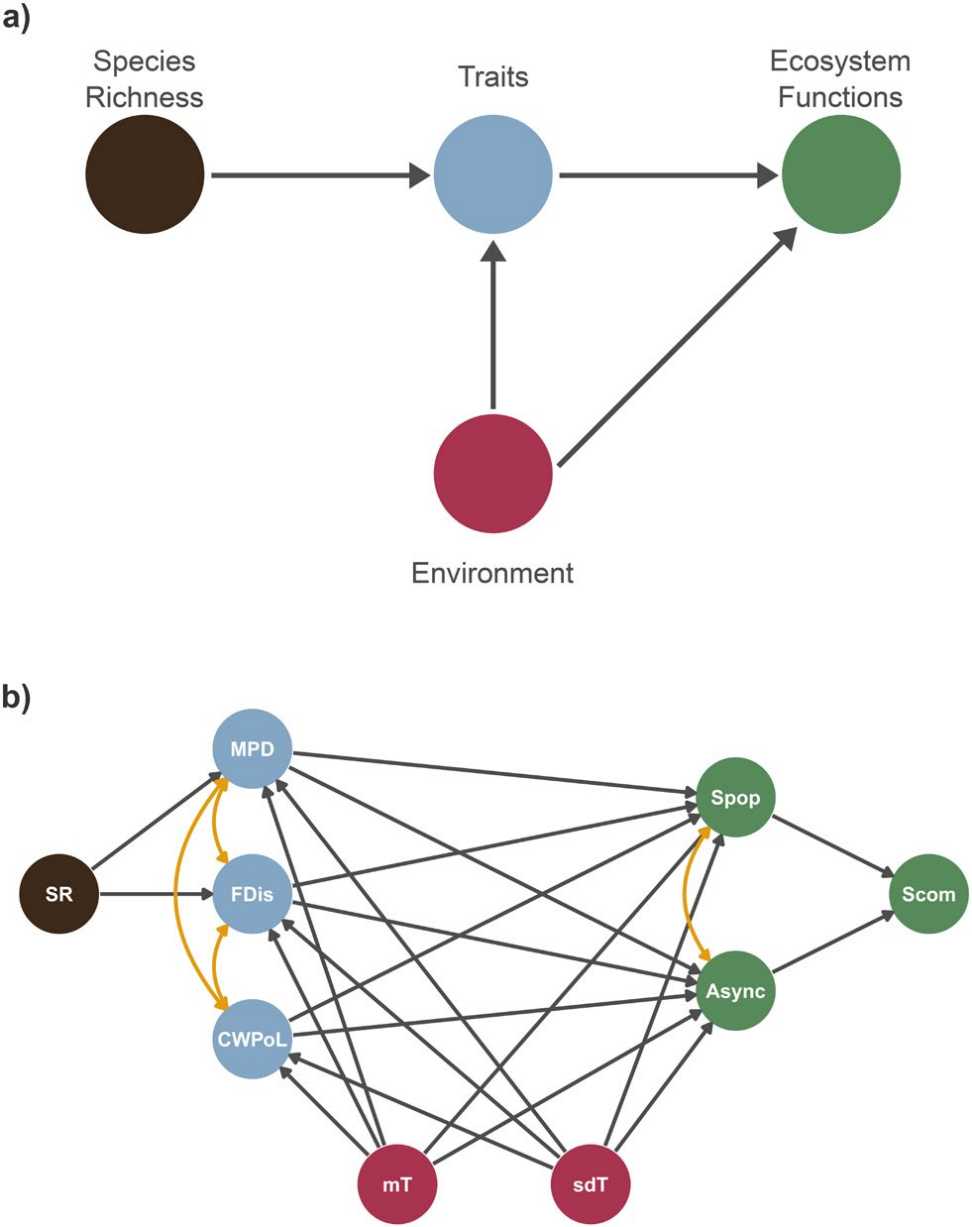
Directed acyclic graph (DAG) stating the causal relationships we expect among species richness, traits, environment, and stability metrics. (a) shows the standard model of causal relationship expected for biodiversity‐stability relationships. (b) shows the causal relationship expected given the variables we used to represent each part of the standard model in (a). Black lines show causal effects and orange lines show correlation terms included in the model. Async = Asynchrony (φ), CWPoL = community weighted mean of pace of life, FDis = functional dispersion, MPD = Mean Phylogenetic Distance, mT = temporal mean of a temperature variable (GDD or FDD), S_com_ = community stability, sdT = temporal standard deviation of a temperature variable (GDD or FDD), S_pop_ = weighted average population stability, SR = species richness.

First, to test for the statistical dependence of community stability separately on diversity and climatic covariates, we used Spearman correlation tests. Then, we used structural equation modelling (Bollen 1989; Grace 2006) to evaluate how species diversity can affect community stability through the responses of functional diversity to the environment. We use the standard graphical causal model for the biodiversity–ecosystem function relationship proposed by Grace, Loreau, and Schmid (2022), which states that species richness affects functional diversity, which in turn affects ecosystem stability, and that environment directly affects functional traits and ecosystem stability (Figure 2a). We used temporal average of species number to represent richness in each community. Temporal mean and standard deviation of GDD and FDD were used to measure average climatic conditions and their fluctuations.

Functional diversity was included in our models using community weighted pace‐of‐life (CWPoL), functional dispersion (FDis; Laliberté and Legendre 2010) and mean phylogenetic pairwise distance (MPD), used as a proxy for unmeasured trait variation (Tucker et al. 2018). CWPoL was defined as the first principal component (PC1) of the standardised taxon‐specific traits (Figure S1). Across taxa, PC1 of the trait space was associated with life‐history traits (e.g., size and generation length) corresponding to the fast‐slow continuum of species' pace‐of‐life. To have CWPoL values from PC1 representing the same direction of fast‐slow continuum across taxa – that is low CWPoL values represent fast while high CWPoL values represent slow pace of life – when needed, we multiplied the PC1 by −1. FDis was calculated with the same traits using the *fdisp* function of R package FD (version 1.0.12.3, Laliberté, Legendre, and Shipley 2015). FDis measures the spread of species‐specific traits as the weighted average distance of species traits from the centroid of the community trait space. MPD describes the phylogenetic relatedness of the species in a community and was computed with the *mpd* function from R package picante (version 1.8.2., Kembel et al. 2010) using the phylogenetic tree of each taxon. In our analysis, we used the temporal averages of FDis, CWPoL and MPD for each site.

For a more refined description of pathways from biodiversity to stability, we divided community stability into two components representing the stability and asynchrony of populations (Thibaut and Connolly 2013). For each site, we measured the community stability as the inverse of the coefficient of variation of the total abundance in a community (number of individuals for all taxa, except biomass for phytoplankton). Community stability $S_{\mathrm{com}}=\frac{\mu_{\mathrm{com}}}{\sigma_{\mathrm{com}}}$, where $\mu_{\mathrm{com}}$ is the temporal mean of community abundance and $\sigma_{\mathrm{com}}$ is the temporal standard deviation of community abundance over the study period. Since community stability is the product of species‐averaged population stability and community asynchrony (Zhao et al. 2022; see also Thibaut and Connolly 2013; Wang and Loreau 2014), we can write that

$$\log S_{\mathrm{com}}=\log\phi +\log S_{\mathrm{pop}}$$

where $\phi =\Sigma \sigma_{j}/\sigma_{\mathrm{com}}$ is the community asynchrony and $S_{\mathrm{pop}}=1/\Sigma \frac{\mu_{j}}{\mu_{\mathrm{com}}}\frac{\sigma_{j}}{\mu_{j}}$ is the species‐averaged population stability (Figure 2b). Here, $\mu_{j}$ and $\sigma_{j}$ are the temporal population mean and standard deviation.

For each taxon, we fitted two structural equation models (SEM) corresponding to Figure 2b using the piecewiseSEM package (version 2.3.0, Lefcheck 2016): one using GDD and the other using FDD as environmental variables. We evaluated the goodness‐of‐fit of each model using the Fisher's C and Chi‐square statistics. Fisher's C tests if the topology of the causal model is supported by the data (Shipley 2004, 2009), while the Chi‐square statistics test if the causal model differs from a saturated model where all the variables are connected (Shipley and Douma 2020). We considered that the causal model (Figure 2b) was supported only when both statistics presented p‐values > 0.05. When the model was not supported, we were guided by the d‐separation test (Shipley 2004) results to modify the topology of the model by adding new arrows of causal relationship, or a correlation term between variables, based on what was most likely needed. We then re‐evaluated the goodness‐of‐fit. Finally, we ranked the models (GDD vs. FDD as environmental variables) for each taxon using the full‐model AIC statistics, which enable comparing models with different topologies, parameter estimates of the SEM and distributional assumptions of the submodels of the piecewise SEM (Shipley and Douma 2020). For the best‐fit models, we also calculated indirect effect sizes (EfS) for each predictor variable by multiplying the direct effects along each path (Lefcheck 2016).

## 3 Results

**FIGURE 3:**
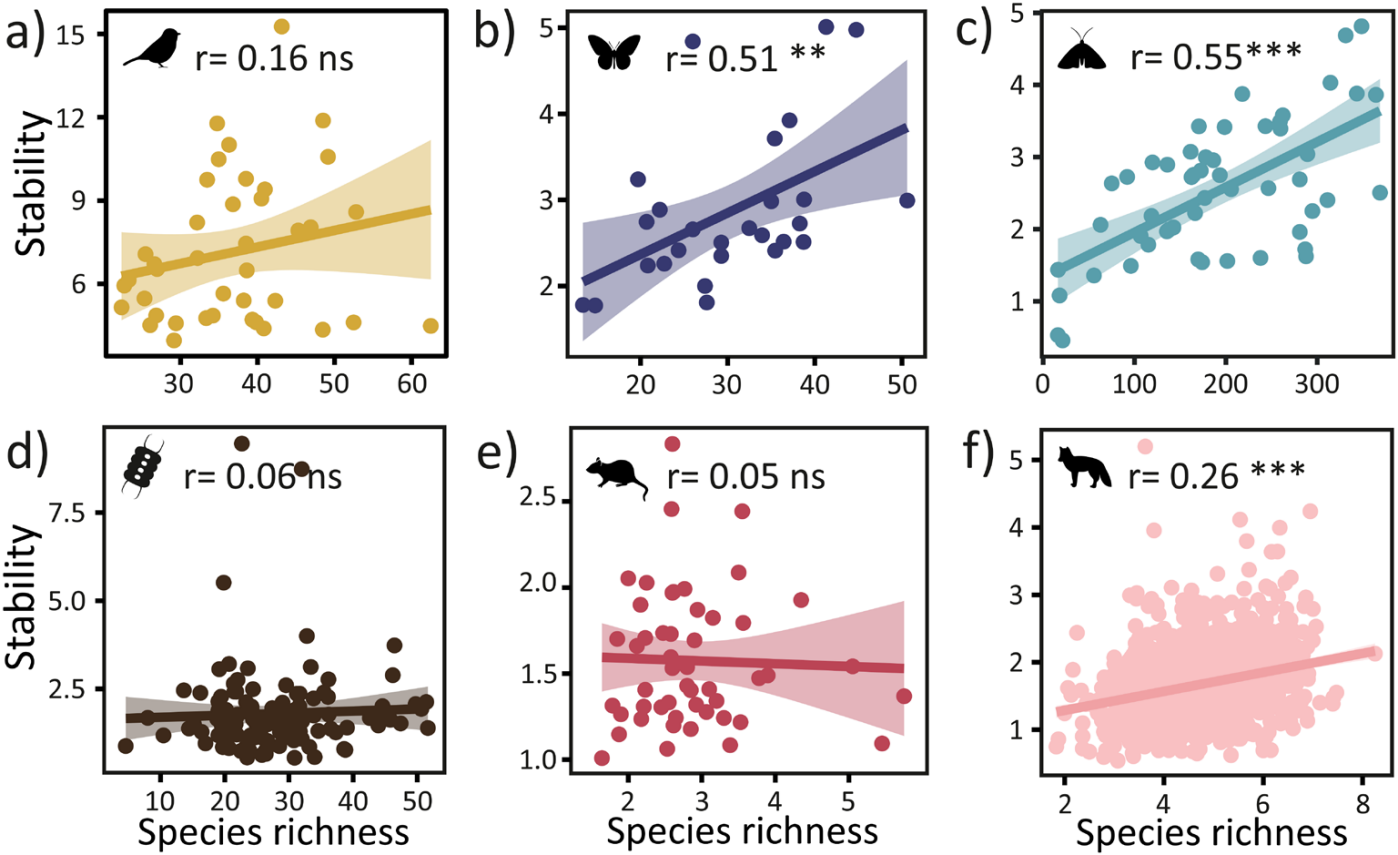
The relationship between community stability and site‐specific species richness in different species groups (a–f). The trend line visualises the linear relationship between the variables and correlation is reported using the Spearman correlation coefficient (*r*). Statistical significance is marked with asterisk (*p < 0.05, **p < 0.01, ***p < 0.001, ns = not significant).

We found the hypothesised positive relationship between species richness and community stability, but its strength varied among the taxa (Figure 3). Positive diversity–stability relationship was observed also with functional diversity metrics (FDis and MPD), whereas the effect of CWPoL on stability was weak across taxa (Figure S2a–c). The richness‐stability relationship was strongest in large mammals, butterflies and moths (*r* = 0.26, 0.51 and 0.55, respectively; Figure 3.). In small mammal and phytoplankton communities' stability was more related to MPD (*r* = 0.26 and *r* = 0.29) and for birds FDis (*r* = 0.2) showed the greatest effect. However, statistical significance of these bivariate relationships varied (Figure S2a,b). Mean GDD and FDD were directly linked to stability with a positive effect in birds, moths and large mammals but, overall, the effect of environmental variables was minor (Figure S2d–g).

**FIGURE 4:**
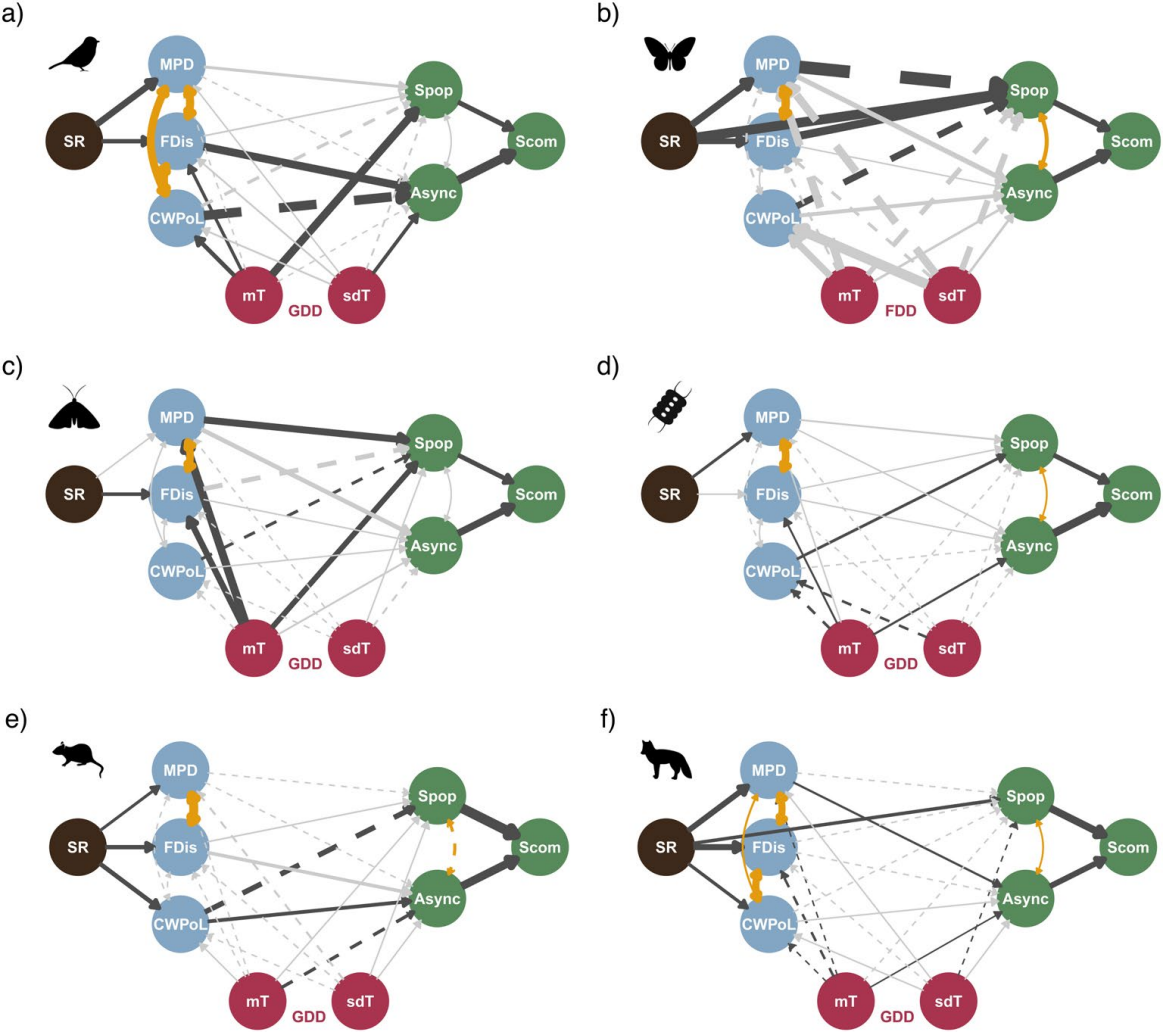
Best fitted structural equation models for each taxon. Black arrows show significant effect, while grey arrows show non‐significant effect. Curved and yellow double‐headed arrows show the significant correlated error terms. Arrow width represents the effect size; solid arrows represent a positive effect, and dashed arrows show a negative effect. The coloured circles follow the standard model components shown in Figure 1a, where black refers to species richness, blue to traits, red to environment and green to ecosystem function. Async = Asynchrony, CWPoL = community weighted pace of life, FDD = Freezing Degree days, FDis = Functional dispersion, GDD = Growing Degree Days, MPD = Mean Phylogenetic Distance, mT = temporal mean of temperature, S_com_ = community stability, sdT = temporal standard deviation of temperature, S_pop_ = weighted average population stability, SR = species richness.

Structural equation modelling revealed that diversity is related to community stability through population stability and asynchrony, but the pathways behind these relationships varied among taxa (Figure 4). Species richness linked to stability indirectly (mean indirect Efs 0.04), either via FDis, MPD or CWPoL; however, these links were not always statistically significant. In the SEMs, the diversity metrics (SR, FDis and MPD) which had a significant relationship with the stability metrics (Spop, Async and Scom) showed mostly a positive effect across taxa (mean indirect EfS 0.14, 0.57 and 0.39, respectively), in line with the direct relationships shown in Figure 3 (see also Figure S2a,b). In contrast, CWPoL traits showed mainly negative effects, with average indirect EfS of −0.24, indicating that community stability is more often driven by the dominance of fast rather than slow species in the studied communities. Across taxa, mean temperature was more significantly linked to diversity and stability variables than its standard deviation (mean indirect EfS ~0.07 for both) and for five out of six species groups GDD was more important driver than FDD. Both GDD and FDD models were supported for each taxon, except for birds (Table 2) and showed similar patterns; therefore, we include only the results of the best‐fit model in the main text (see Figures S3–S8 for detailed results for all SEMs, and Figure S9 for the indirect effects).

**TABLE 2:** Goodness‐of‐fit statistics for the Structural Equation Models comparison between the use of Freezing Degree Days (FDD) or Growing Degree Days (GDD) as the environmental variables for each taxon. In each taxon, models are ordered by ΔAICc, bold in model name and *p*‐values show supported models.

Model	Fisher's C (df, *p*)	*χ* ^2^ (df, *p*)	AICc	ΔAICc
Birds				
FDD	34.121 (18, 0.012)	14.683 (9, **0.100**)	−2372.314	0.000
**GDD**	26.883 (18, **0.081**)	12.123 (9, **0.206**)	−2371.525	0.789
Butterflies				
**FDD**	13.012 (16, **0.672**)	5.452 (8, **0.708**)	−1612.720	0.000
**GDD**	15.630 (16, **0.479**)	6.806 (8, **0.558**)	−1609.331	3.389
Moths				
**GDD**	13.785 (18, **0.743**)	7.231 (9, **0.613**)	−3343.787	0.000
**FDD**	7.193 (18, **0.988**)	3.031 (9, **0.963**)	−3325.279	18.508
Phytoplankton				
**GDD**	7.119 (18, **0.989**)	3.124 (9, **0.959**)	−7313.255	0.000
**FDD**	7.283 (18, **0.988**)	3.132 (9, **0.959**)	−7302.004	11.251
Small Mammal				
**GDD**	23.644 (16, **0.098**)	14.955 (8, **0.06**)	−2917.958	0.000
**FDD**	23.612 (16, **0.098**)	15.02 (8, **0.059**)	−2916.038	1.920
Large Mammal				
**GDD**	9.558 (14, **0.794**)	3.25 (7, **0.861**)	−61409.744	0.000
**FDD**	10.85 (14, **0.698**)	2.554 (7, **0.923**)	−61384.469	25.275

Results suggest that the different facets of diversity seem to influence community stability primarily by altering population stability. However, asynchrony had a slightly larger direct impact on overall community stability (mean direct EfS for Async 0.75 and for S_pop_ 0.62 respectively). The specific pathways linking diversity, traits and environment to stability differed among species groups.

In bird communities, FDis was linked to community stability via asynchrony (indirect EfS 0.68). CWPoL was also linked to asynchrony showing a negative effect on community stability (indirect EfS −0.81), which suggests that a faster pace‐of‐life increases stability (Figure 4a, Figure S9a). Moreover, bird population and community stability were strongly mediated by temperature variables (both its mean and temporal variation) indicating that warmer summers increase stability, but only GDD model showed support for the causal relationships (Table 2).

For butterflies the FDD model fit better, even though temperature variables did not show any significant paths (Figure 4b). In butterfly communities, species richness and FDis had a strong positive effect on population stability (direct EfS 0.96 and 0.8 respectively), whereas none of the variables used seemed to link to asynchrony. A similar pattern was observed also in moths as the diversity variables predicted population stability better than asynchrony, although the effect of asynchrony on community stability was larger (Figure 4c). Warmer summers, as well as faster and phylogenetically more diverse moth communities, increased population and community stability (indirect EfS 0.25, −0.14 and 0.32, respectively, Figure S9c).

Conversely, in phytoplankton communities, stability increased with the presence of slower species (indirect EfS 0.13), but none of the diversity metrics was linked to community stability (Figure 4d, Figure S9d). In small mammal communities, species richness was linked to FDis, MPD and CWPoL, but only CWPoL was linked both to population stability and asynchrony with indirect effects of −0.5 and 0.29 on community stability (Figure 4e, Figure S9e).

In large mammal communities, species richness linked indirectly to community stability via MPD and asynchrony (indirect EfS 0.07), but also more directly via population stability (indirect EfS 0.25), whereas FDis and CWPoL showed no effect on community stability (Figure 4f, Figure S9f). Temperature variables had a minor effect on community stability in phytoplankton, small mammal and large mammal communities.

## 4 Discussion

Understanding the diversity–stability relationship in natural communities is of utmost importance, as all dimensions of natural biodiversity are undergoing rapid change, and the stability of these communities underpins the reliable delivery of vital ecosystem functions and services (Cardinale et al. 2012). Here, we provide evidence that the diversity–stability relationship is often mediated by functional traits in natural communities across different taxonomic groups (cf. Grace, Loreau, and Schmid 2022), but with taxon‐dependent underpinnings. By using long‐term species' monitoring data across a large spatial extent, our findings support previous studies (see e.g., Downing, Brown, and Leibold 2014, Craven et al. 2018, Zhao et al. 2022, but see e.g., Evans et al. 2022, 2023, Wisnoski et al. 2023 for natural communities). However, our approach enables us to identify how the pathways differ among taxa.

Richer communities are more likely to fluctuate asynchronously, as higher species number can increase the asynchrony of communities, as predicted by the so‐called portfolio effect (Thibaut and Connolly 2013; Zhao et al. 2022). However, our models did not support this mechanism as no direct link was observed between species richness and asynchrony. A direct link between species richness and stability components existed in butterflies and large mammals but, in these taxa, richness was linked to population stability not asynchrony. Also, the other diversity facets were more often related to population stability across taxa, even though asynchrony showed larger direct effect on community stability. This could indicate that compensatory dynamics and statistical averaging effects, through which asynchrony promotes stability (Zhao et al. 2022), can also act independently of diversity.

We found that stability is more related to functional diversity and community composition. Functional diversity may increase community stability via several mechanisms, which can drive the observed relationships between functional dispersion, mean phylogenetic distance and the stability components. First, strong competition between species can destabilise communities by increasing the amplitude of population fluctuations, causing large temporal variation in total individual abundances (or biomass) (Loreau and de Mazancourt 2013). Respectively, in functionally diverse communities the average population stability can increase, as species occupying different niches reduce interspecific competition (Loreau and de Mazancourt 2008). Second, temporal stability can be promoted through compensatory dynamics as species respond differently to abiotic and biotic conditions depending on their life‐history strategy and associated traits (Loreau and de Mazancourt 2013; de Bello et al. 2021). Third, communities with higher trait diversity can support stability through a change in dominant species, as species with different life strategies can replace one another maintaining similar relative abundance, for example, when faced with environmental perturbations (de Bello et al. 2021). Increase in functional diversity was linked to stability in bird, large mammal, butterfly and moth communities, but the extent to which the presented ecological mechanisms drive the observed relationship in each taxon remains speculative. We included MPD in our analyses as a proxy for unmeasured trait variation and shared evolutionary history to account for the fact that changes in communities are often non‐random with respect to phylogeny (Cadotte, Albert, and Walker 2013; Uchida, Hiraiwa, and Cadotte 2019). Therefore, its effects on community stability can differ from those of functional dispersion. Moreover, depending on the mode of trait evolution, the extent to which phylogenetic diversity metrics capture unmeasured trait differences between species can vary, potentially leading to differences in responses (Tucker et al. 2018).

In addition to functional diversity metrics, we found community pace‐of‐life to often affect community stability, but with varied causal paths across taxa. Our results suggest that both slower and faster communities can increase population stability and asynchrony, indicating that different mechanisms drive the relationship between species' pace‐of‐life and stability. Species with a slower pace‐of‐life are often more resistant to environmental fluctuations and hence capable of maintaining stable communities through buffered population growth. In contrast faster species are less resistant to change but recover more rapidly after disturbances (Craven et al. 2018, Capdevila et al. 2020). Both slow and fast species can increase stability via insurance effect in which functionally redundant species can replace one another following some exceptional perturbation (de Bello et al. 2021). In our models, higher asynchrony and population stability were mostly associated with faster communities (e.g., in birds and butterflies), yet in phytoplankton communities, higher population stability was linked to slower communities, which can indicate the stabilising effect of functionally conservative (slow) dominant species. Summarising the effects of trait‐related variables on stability, our results suggest that species richness promotes functional diversity and, in turn, stability. However, the dynamics differ among taxa, with fast or slow species driving community fluctuations through either population stability or asynchrony.

Even though environmental conditions are known to shape species ranges (Antão et al. 2022, Hällfors et al. 2023), diversity (Marjakangas et al. 2022), functional composition (Bosco et al. 2023) and community stability (Evans et al. 2022; Tredennick et al. 2017), in our models, temperature showed large effect on community stability only in birds and moths. Overall, mean temperature was a more important driver of stability than fluctuations in temperature. Additionally, higher functional diversity and community stability were mostly related to warmer summer and winter conditions (depicted by GDD and FDD respectively), which indicates increase both in diversity and stability towards southern Finland. Although we focused here on temperature due to its known importance in driving communities' variability across taxa (Bowler et al. 2017), species respond to many other environmental conditions as well and vary in their sensitivity to these (Lawson et al. 2015; Mills, Oliver, and Bradbury 2017). Therefore, other environmental dimensions may mediate taxon‐specific responses that underlie the diversity–stability relationship.

Regarding the observed diversity–stability relationships and their drivers we emphasise that our results depend on the used data and therefore present patterns in the observed communities. Even though we utilise high quality systematic monitoring data and have substantial temporal coverage, the observed communities capture only a subset of the total communities, as is the case with any observational ecological data based on sampling. However, given the systematic nature of the spatiotemporal sampling and the fact that we are not focusing on the effect of individual species but the community composition, we don't expect our results to be affected by structured biases in the collected data.

To conclude, our results support the understanding that species richness affects community stability indirectly via changes in functional diversity and composition (Craven et al. 2018; de Bello et al. 2021; Grace, Loreau, and Schmid 2022), which affect population stability and asynchrony (Thibaut and Connolly 2013). Long‐term monitoring data on six taxon groups, together with functionally analogous traits, enabled us to demonstrate that diversity begets stability across a wide array of taxa in natural communities along a broad latitudinal gradient. Our study highlights the need for both systematic monitoring data and high‐quality trait data to unravel processes underpinning key ecosystem functions. Our results further highlight how sensitive vital ecosystem functions are to ongoing anthropogenic global change and the resulting biodiversity crisis.

## Author Contributions

A. V. R. and T. R. wrote the first draft of the manuscript with shared lead authorship and conducted the analyses. A. V. R., T. R., M. M. J., M. S., J. V. and A.‐L. L. conceptualised the study and had equal major contribution to the initial research design and formulation of the manuscript. I.‐M. H., O. H., E. K., M. K., A. Lehikoinen, A. Lindén, H. P., J. P., P. S., A. S. and K. V. provided the data. M. S., J. V. and A.–L. L. have jointly supervised this work. All authors contributed to the writing of the manuscript.

### Peer Review

The peer review history for this article is available at https://www.webofscience.com/api/gateway/wos/peer‐review/10.1111/ele.70003.

## Supporting information


Data S1.

## Data Availability

The data and code necessary to reproduce this work are available at https://doi.org/10.5281/zenodo.13927807.
