# Supplementary material for: Cross‐Taxa Analysis of Long‐Term Data Reveals a Positive Biodiversity‐Stability Relationship With Taxon‐Specific Mechanistic Underpinning
Source: Ecol Lett. 2025 Apr 3;28(4):e70003. doi: 10.1111/ele.70003 (PMC11966686; doi:10.1111/ele.70003)
Supplement: Supplementary file 1 — Data S1. [file ELE-28-0-s001.pdf]

Supporting Information for “Cross-taxa analysis of long-term data reveals a positive biodiversity-stability relationship with taxon-specific mechanistic underpinning”

Table S1. Traits used to describe the pace-of-life and functional diversity in each taxon.

Taxon	Trait name	Description	Type	Function
Birds	Migration	Species migratory status: resident, short-distance migrant or long-distance migrant.	Categorical	Dispersal
	Generation length	Species generation length in years. (Log-transformed)	Continuous	Reproduction
	Body mass	Species body mass in grams. (Log-transformed)	Continuous	Growth
	Maximum brood	Maximum number of broods per year (Log-transformed).	Continuous	Reproduction
	Feeding	Diet category: insectivore, carnivore, herbivore, omnivore or mixed.	Categorical	Resource use
Butterflies and moths	Wintering life stage	The life history stage in which a species hibernates: egg, larvae, pupa or adult.	Categorical	Survival
	Voltinism	The number of generations a species has in a year: univoltine (one), bivoltine (two), multivoltine (three or more).	Categorical	Reproduction
	Host usage	The range of hostplants that a species can use: monophagous (one species), oligophagous (several species within one genus or family), polyphagous (several species and families.)	Categorical	Resource use
	Wingspan	Distance between the apices of the two forewings measured in millimetres. (Log-transformed)	Continuous	Dispersal and growth
Small and large mammals	Generation length	Average age of parents in days. (Log-transformed)	Continuous	Reproduction
	Litter size	Number of offspring born per litter per female.	Continuous	Reproduction
	Diet breadth	Number of different food elements a species consumes.	Continuous	Resource use
	Body mass	Species body mass in grams. (Log-transformed)	Continuous	Growth
	Dispersal distance	Distance in kilometers a species travels between the birth and breeding site. (Log-transformed)	Continuous	Dispersal
Phytoplankton	Nitrogen fixation	Species ability to fix nitrogen, which can provide a competition advantage.	Binary	Resource use
	Motility	Species ability to move and migrate.	Binary	Dispersal
	Colony formation	Larger cell sizes (i.e., ability to form colonies) decrease grazing pressure and reduce mortality.	Binary	Survival
	Cell volume	Species size measured in micrometers cubed.	Continuous	Growth

Table S2. References of bird trait data. Full reference is provided when first mentioned and after that only citation is shown.

**Trait**	**References**
Migratory status	Valkama, J. Saurola, P. Lehikoinen, A. Lehikoinen, E. Piha, M. Sola, P. and Velmala, W. (2014). *The Finnish Bird Ringing Atlas.* Vol. II. Finnish Museum of Natural History and Ministry of Environment, Helsinki.
Generation length	
Body mass	Cramp, S., Simmons, K.E.L., Perrins, C.M. (1977-1994). *Handbook of the Birds of Europe, the Middle East and North Africa*: Birds of the Western Palearctic. Oxford University Press, Oxford.<br><br>Piha, M. & Lehikoinen, E. (2016). Body mass and wing length of birds based on the Finnish ringing database — Part 1: non-corvid passerines. *Linnut-vuosikirja 2015*:142-151.
Maximum brood	Cramp et al. (1977-1994)<br><br>Solonen, T. (1985). *Suomen Linnusto*: Esiintyminen ja perusbiologiaa, 280 p. Lintutieto.
Feeding	Brommer, J. (2008). Extent of recent polewards range margin shifts in Finnish birds depends on their body mass and feeding ecology. *Ornis Fennica* 85: 105-117.<br><br>Cramp et al. (1977-1994)<br>Wilman, H., Belmaker, J., Simpson, J., de la Rosa, C., Rivadeneira, M. M. & Jetz, W. (2014). EltonTraits 1.0: Species-level foraging attributes of the world's birds and mammals. *Ecology* 95:2027. http://dx.doi.org/10.1890/13-1917.1

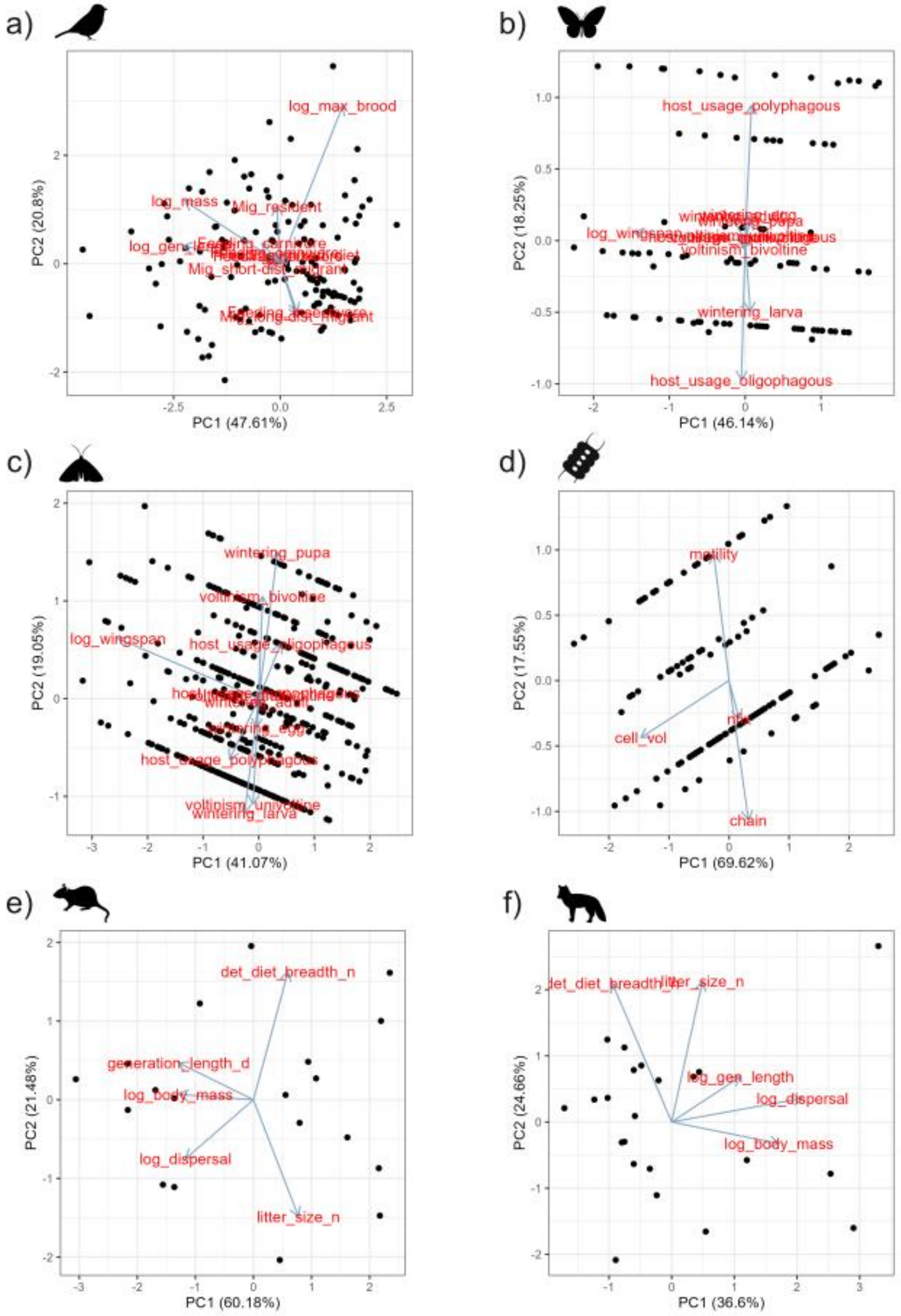

Figure S1 – Principal component analysis of the functional trait variation in each taxonomic group: a) birds, b) butterflies, c) moths, d) phytoplankton, e) small mammals, f) large mammals.

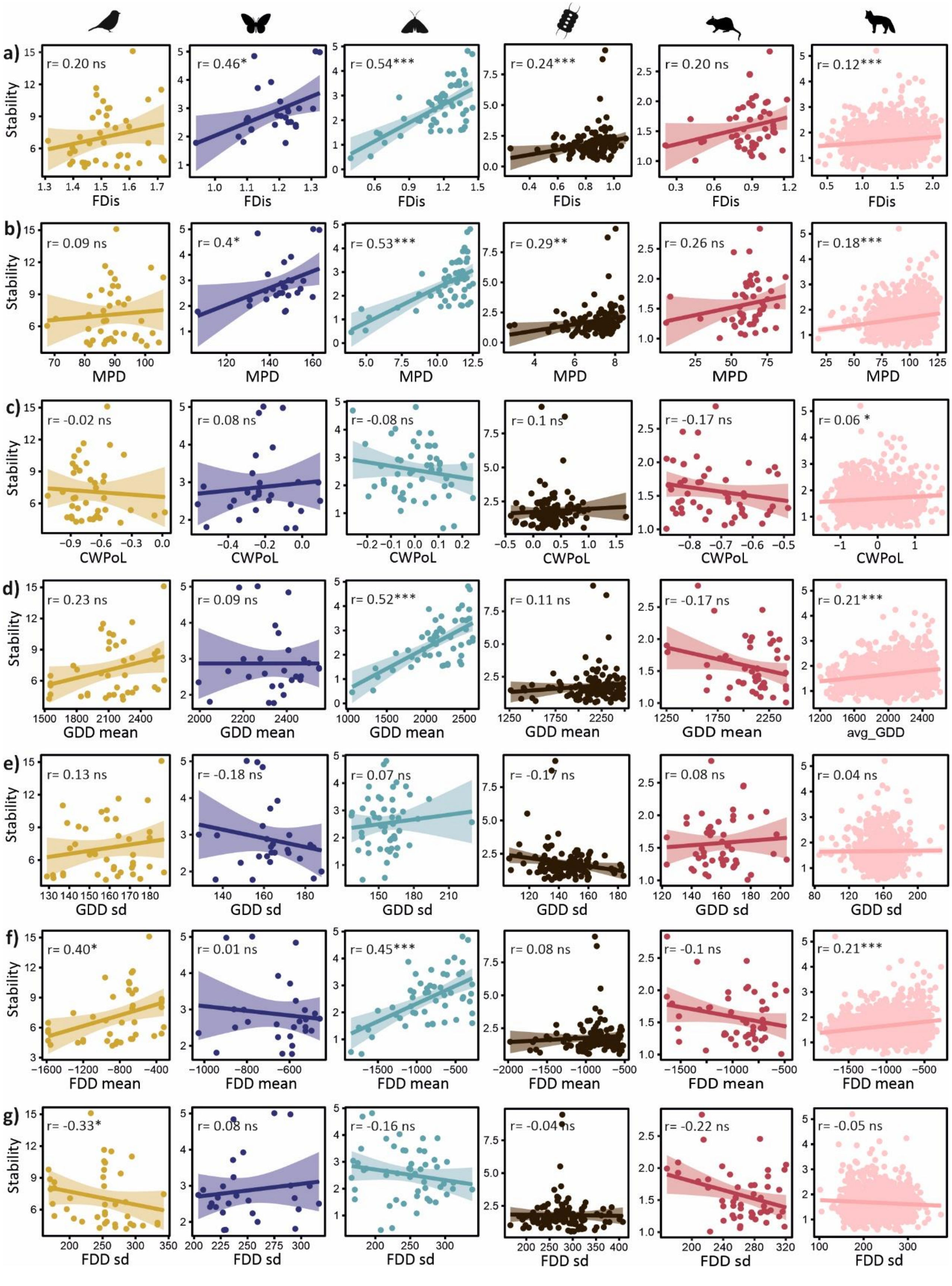

Figure S2. The relationships between community stability and functional dispersion (FD; a), mean phylogenetic distance (MPD; b), community weighted pace-of-life traits (CWPoL; c) as well as mean and standard deviation of growing degree (GDD; d-e) and freezing degree days (FDD; f-g) across taxa. Trend line visualized the linear relationship between the variables and correlation is reported using the Spearman correlation coefficient (r). Statistical significance is marked with asterisk (* = p<0.05, ** = p<0.01, *** = p<0.001, ns = not significant).

Birds (GDD)

Fischer's C = 26.883; df = 18; p−value = 0.081
Chi−square = 12.123; df = 9; p−value = 0.206
AICc = −2371.525

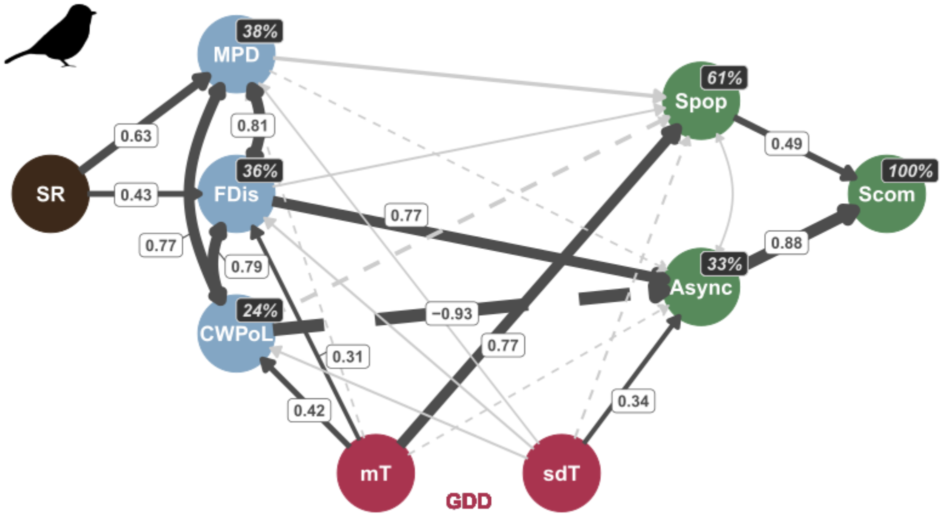

Birds (FDD)

Fischer's C = 34.121; df = 18; p−value = 0.012
Chi−square = 14.683; df = 9; p−value = 0.1
AICc = −2372.314

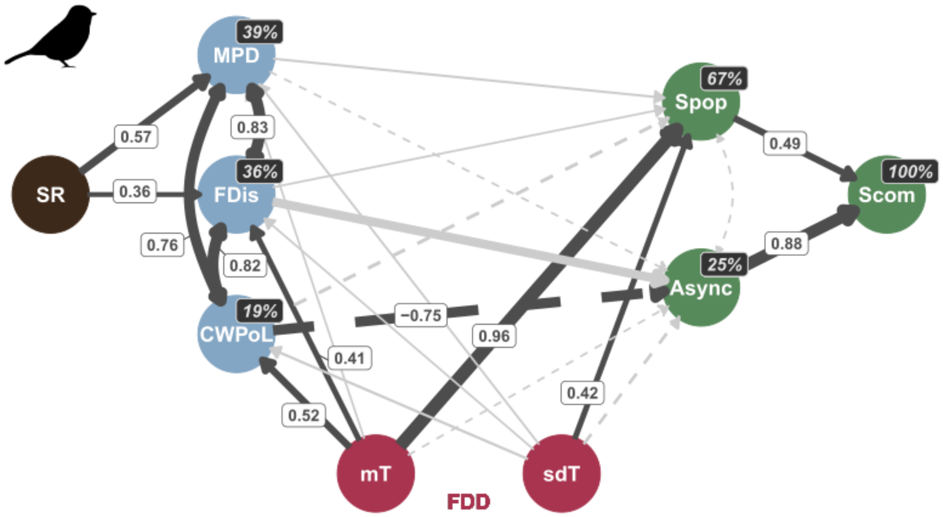

Figure S3 - Structural equation models for bird data using growing degree days (GDD) and freezing degree days (FDD) as temperature variable. Straight lines represent causal paths while curved lines represent correlation terms. Black arrows show significant effect – for which coefficients are shown in a white background box – while grey arrows show non-significant effect. Arrow width represents the effect size; solid arrows represent a positive effect and dashed arrows show a negative effect. Boxes with black background show the R^2 (%) for each response variable in the model. Coloured circles follow the standard model components where black refers to species richness, blue to traits, red to environment and green to ecosystem function. SR = species richness, FDis = functional dispersion, MPD = mean phylogenetic distance, CWPoL = community weighted pace-of-life, mT = temporal mean of temperature, sdT = temporal standard deviation of temperature, FDD = freezing degree days, GDD = growing degree days, S_{pop} = weighted average population stability, Async = asynchrony, S_{com} = community stability.

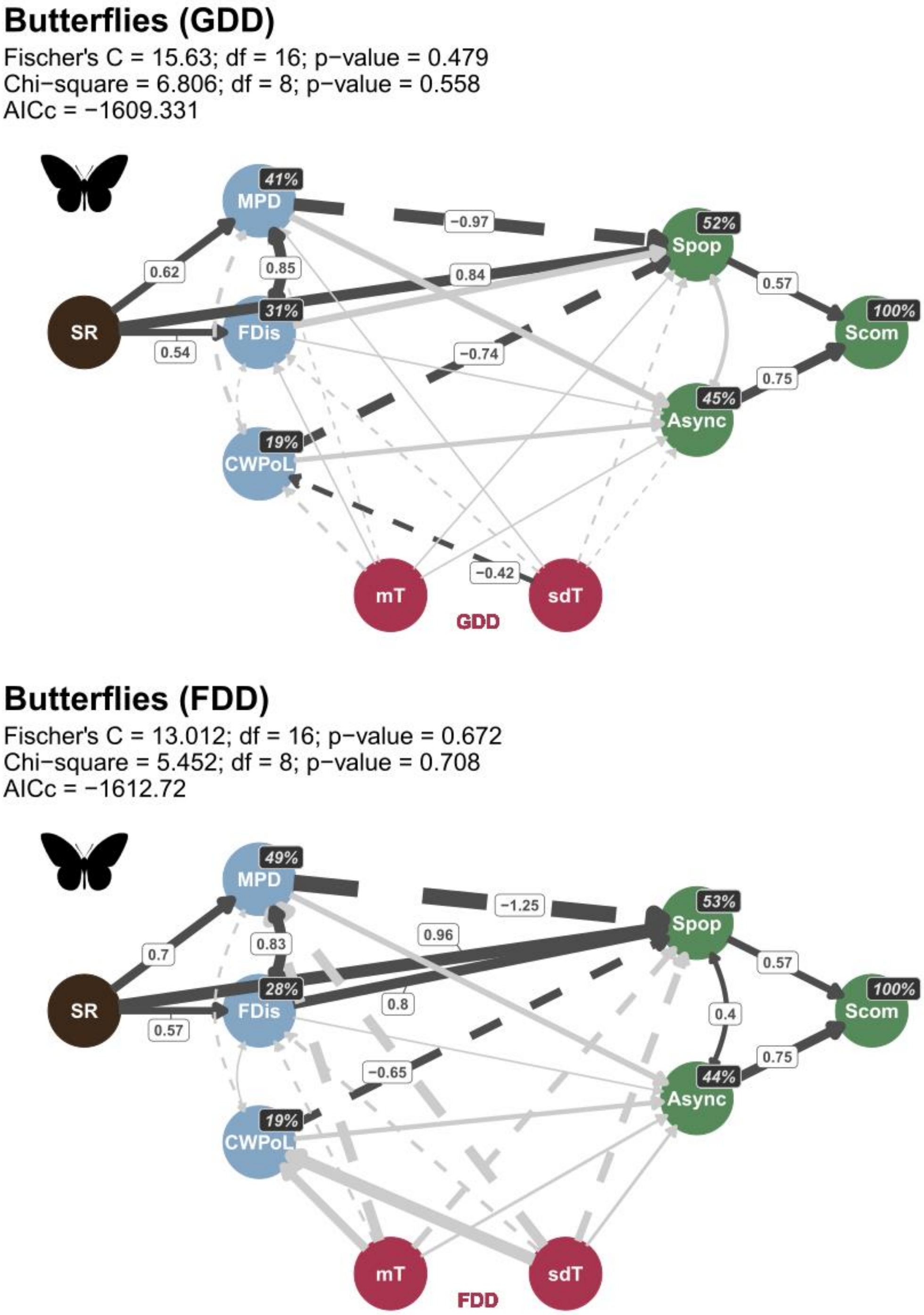

Figure S4 - Structural equation models for butterfly data using growing degree days (GDD) and freezing degree days (FDD) as temperature variable. Straight lines represent causal paths while curved lines represent correlation terms. Black arrows show significant effect – for which coefficients are shown in a white background box – while grey arrows show non-significant effect. Arrow width represents the effect size; solid arrows represent a positive effect and dashed arrows show a negative effect. Boxes with black background show the R^2 (%) for each response variable in the model. Coloured circles follow the standard model components where black refers to species richness, blue to traits, red to environment and green to ecosystem function. SR = species richness, FDis = functional dispersion, MPD = mean phylogenetic distance, CWPoL = community weighted pace-of-life, mT = temporal mean of temperature, sdT = temporal standard deviation of temperature, FDD = freezing degree days, GDD = growing degree days, S_{pop} = weighted average population stability, Async = asynchrony, S_{com} = community stability.

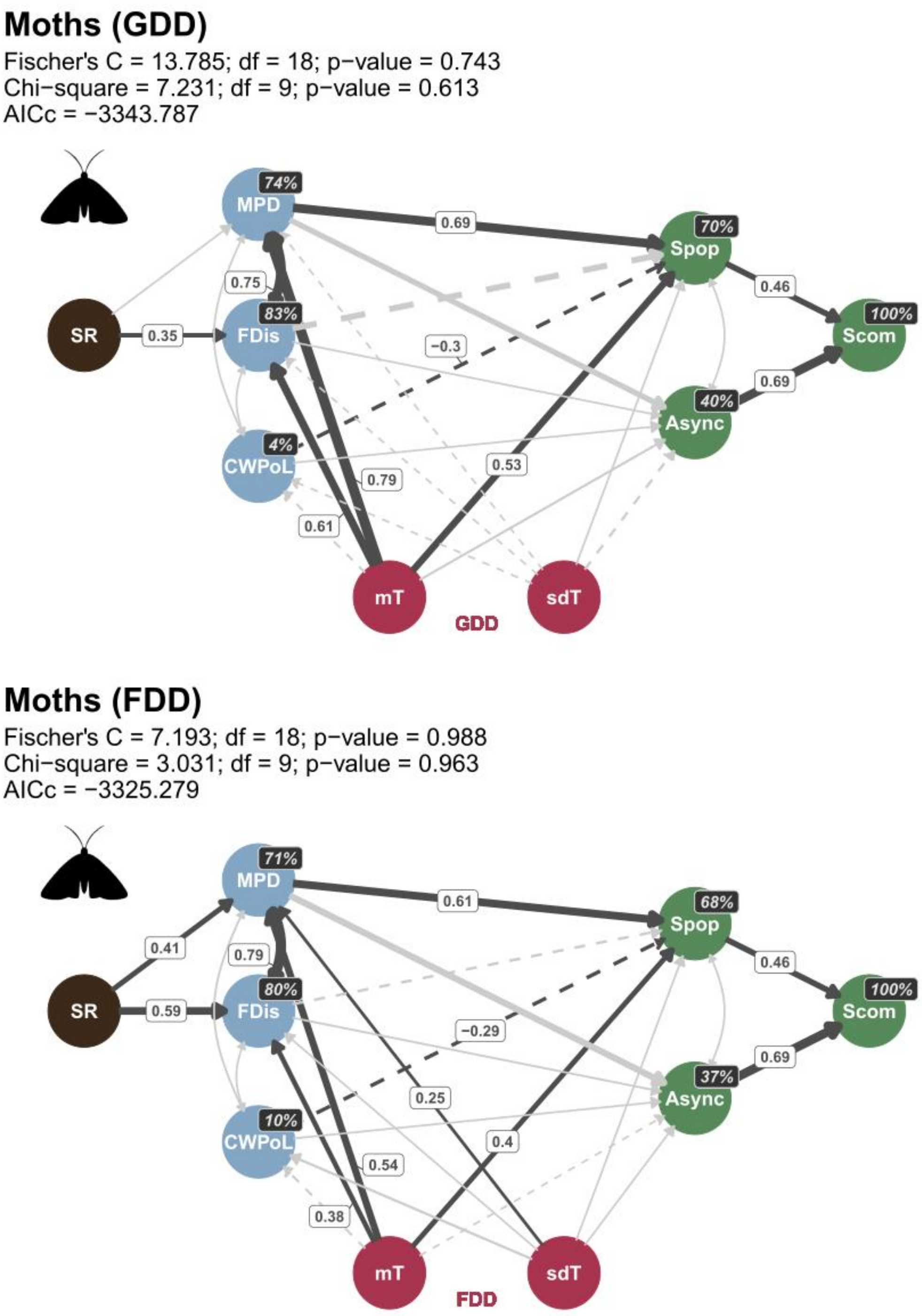

Figure S5 - Structural equation models for moth data using Growing Degree Days (GDD) and Freezing Degree Days (FDD) as temperature variable. Straight lines represent causal paths while curved lines represent correlation terms. Black arrows show significant effect – for which coefficients are shown in a white background box – while grey arrows show non-significant effect. Arrow width represents the effect size; solid arrows represent a positive effect and dashed arrows show a negative effect. Boxes with black background show the R^2 (%) for each response variable in the model. Boxes with black background show the R^2 (%) for each response variable in the model. Coloured circles follow the standard model components where black refers to species richness, blue to traits, red to environment and green to ecosystem function. SR = species richness, FDis = functional dispersion, MPD = mean phylogenetic distance, CWPoL = community weighted pace-of-life, mT = temporal mean of temperature, sdT = temporal standard deviation of temperature, FDD = freezing degree days, GDD = growing degree days, S_{pop} = weighted average population stability, Async = asynchrony, S_{com} = community stability.

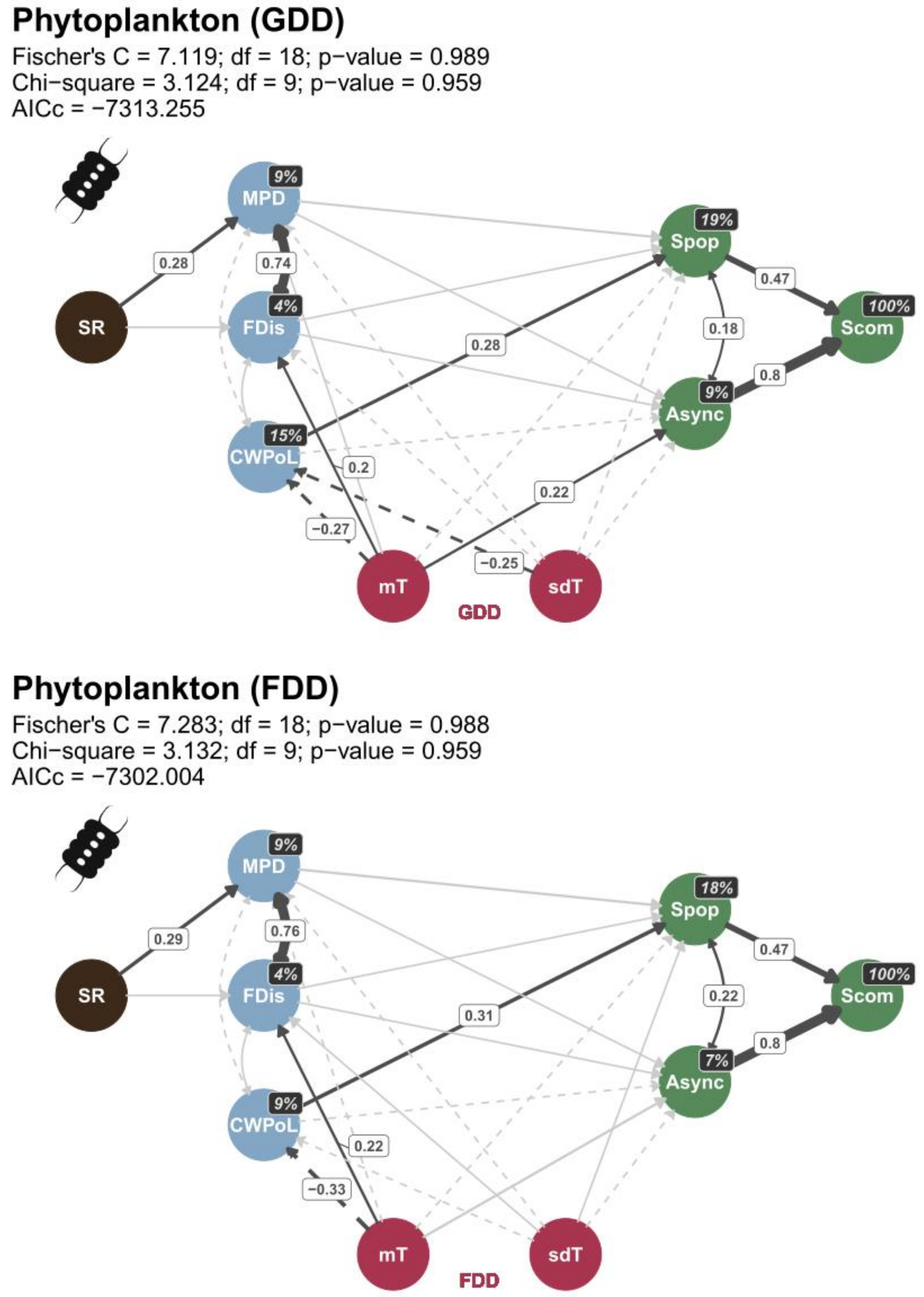

Figure S6 - Structural equation models for phytoplankton data using Growing Degree Days (GDD) and Freezing Degree Days (FDD) as temperature variable. Straight lines represent causal paths while curved lines represent correlation terms. Black arrows show significant effect – for which coefficients are shown in a white background box – while grey arrows show non-significant effect. Arrow width represents the effect size; solid arrows represent a positive effect and dashed arrows show a negative effect. Boxes with black background show the R^2 (%) for each response variable in the model. Coloured circles follow the standard model components where black refers to species richness, blue to traits, red to environment and green to ecosystem function. . SR = species richness, FDis = functional dispersion, MPD = mean phylogenetic distance, CWPoL = community weighted pace-of-life, mT = temporal mean of temperature, sdT = temporal standard deviation of temperature, FDD = freezing degree days, GDD = growing degree days, S_{pop} = weighted average population stability, Async = asynchrony, S_{com} = community stability.

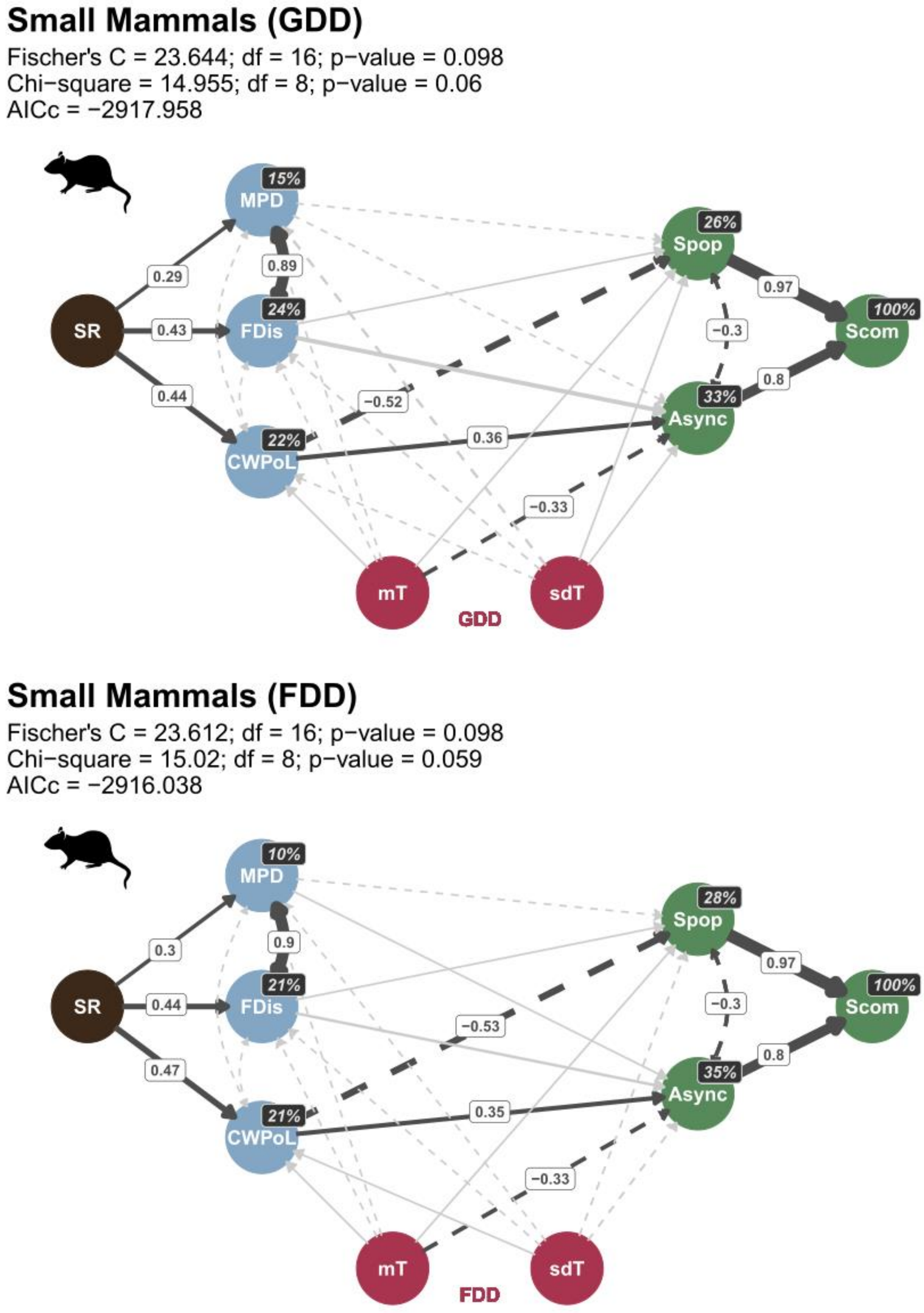

Figure S7 - Structural equation models for small mammal data using Growing Degree Days (GDD) and Freezing Degree Days (FDD) as temperature variable. Straight lines represent causal paths while curved lines represent correlation terms. Black arrows show significant effect – for which coefficients are shown in a white background box – while grey arrows show non-significant effect. Arrow width represents the effect size; solid arrows represent a positive effect and dashed arrows show a negative effect. Boxes with black background show the R^2 (%) for each response variable in the model. Coloured circles follow the standard model components where black refers to species richness, blue to traits, red to environment and green to ecosystem function. SR = species richness, FDis = functional dispersion, MPD = mean phylogenetic distance, CWPoL = community weighted pace-of-life, mT = temporal mean of temperature, sdT = temporal standard deviation of temperature, FDD = freezing degree days, GDD = growing degree days, S_{pop} = weighted average population stability, Async = asynchrony, S_{com} = community stability.

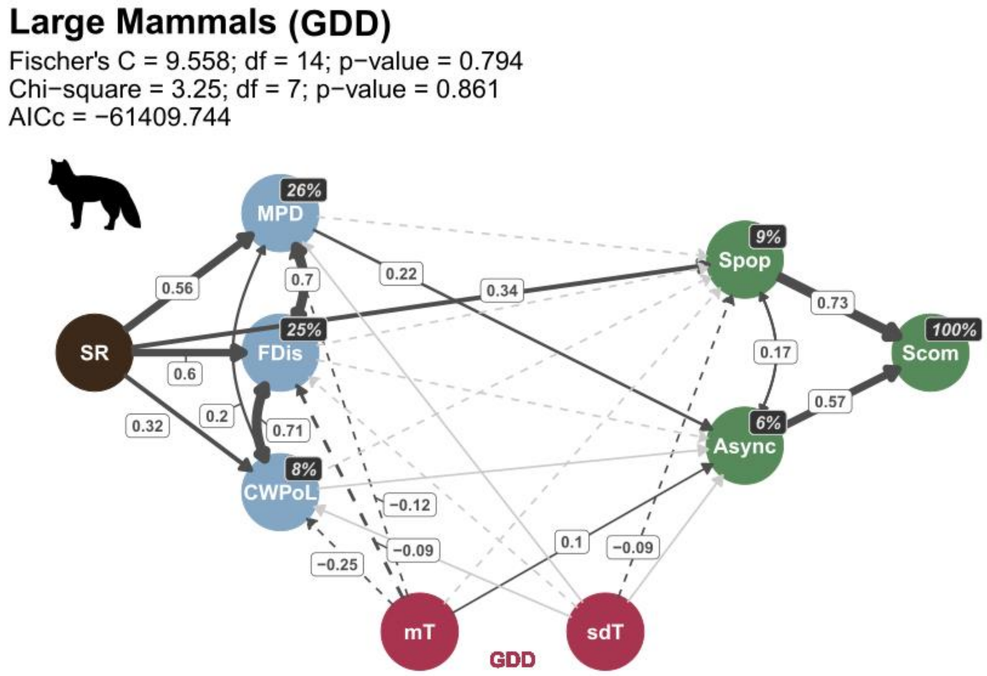

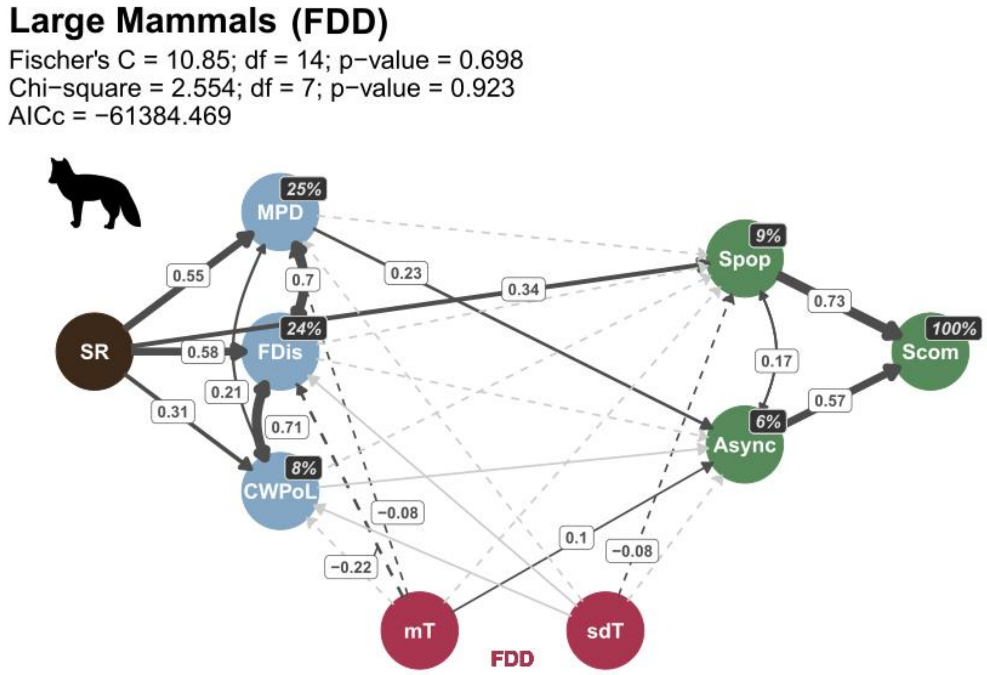

Figure S8 - Structural equation models for large mammal data using Growing Degree Days (GDD) and Freezing Degree Days (FDD) as temperature variable. Straight lines represent causal paths while curved lines represent correlation terms. Black arrows show significant effect – for which coefficients are shown in a white background box – while grey arrows show non-significant effect. Arrow width represents the effect size; solid arrows represent a positive effect and dashed arrows show a negative effect. Boxes with black background show the R^2 (%) for each response variable in the model. Coloured circles follow the standard model components where black refers to species richness, blue to traits, red to environment and green to ecosystem function. SR = species richness, FDis = functional dispersion, MPD = mean phylogenetic distance, CWPoL = community weighted pace-of-life, mT = temporal mean of temperature, sdT = temporal standard deviation of temperature, FDD = freezing degree days, GDD = growing degree days, S_{pop} = weighted average population stability, Async = asynchrony, S_{com} = community stability.

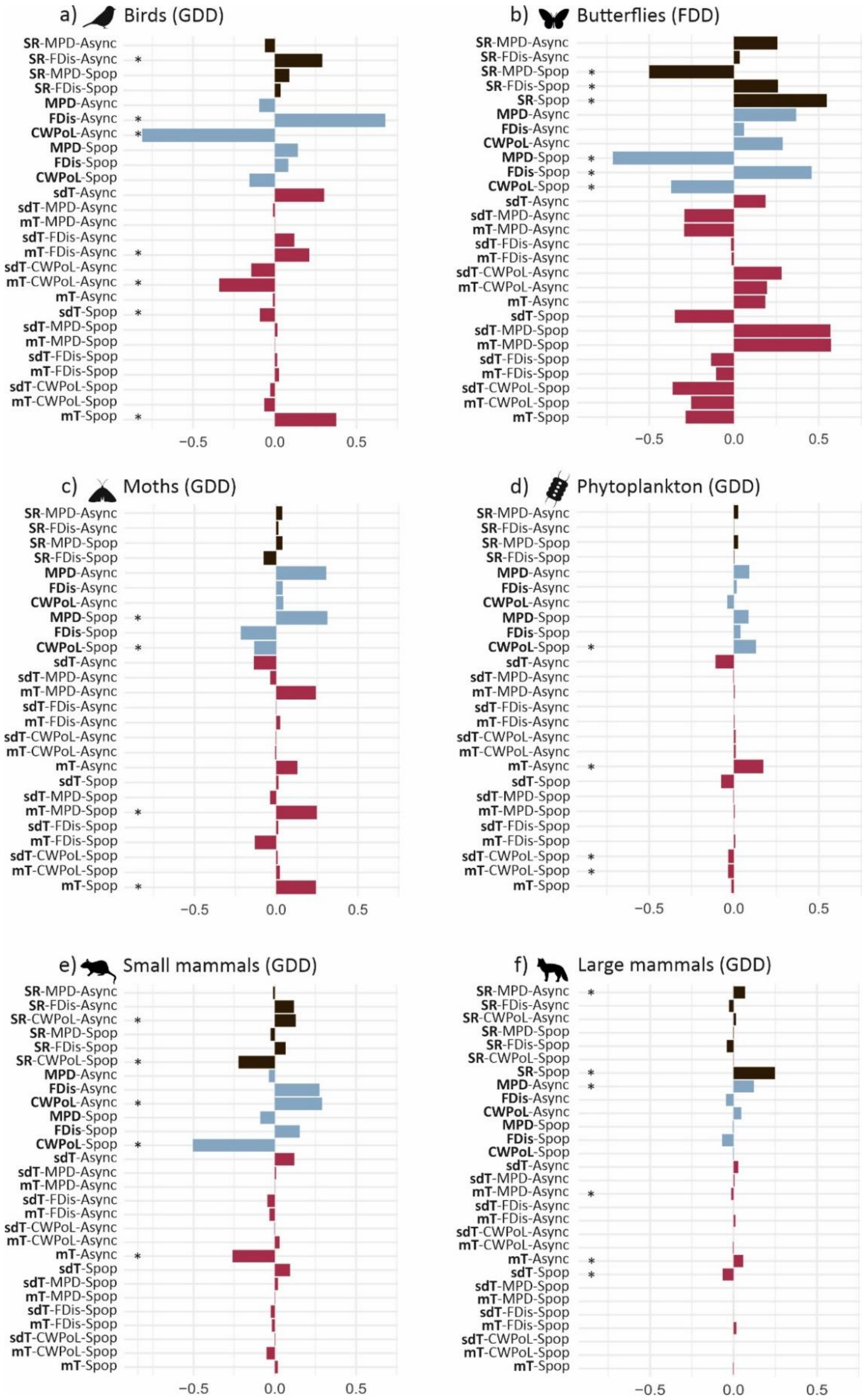

Figure S9. Indirect effects of diversity and environmental variables on community stability in the best fit structural equation model for each taxon. Indirect effects are calculated for each path in a model and plots show both statistically significant (marked with asterisk) and insignificant paths. SR = species richness, FDis = functional dispersion, MPD = mean phylogenetic distance, CWPoL = community weighted pace of life, mT = temporal mean of temperature, sdT = temporal standard deviation of temperature, FDD = freezing degree days, GDD = growing degree days, S_{pop} = weighted average population stability, Async = asynchrony
